# Comparison Between the Effects of Alfentanil, Fentanyl and Sufentanil on Hemodynamic Indices During Rapid Sequence Intubation in the Emergency Department

**DOI:** 10.5812/aapm.14618

**Published:** 2014-01-25

**Authors:** Mahboob Pouraghaei, Payman Moharamzadeh, Hassan Soleimanpour, Farzad Rahmani, Saeid Safari, Ata Mahmoodpoor, Hanieh Ebrahimi Bakhtavar, Robab Mehdizadeh Esfanjani

**Affiliations:** 1 Anesthesiology Research Team, Department of Anesthesiology, Tabriz University of Medical Sciences, Tabriz, Iran; 2 Cardiovascular Research Center, Tabriz University of Medical Sciences, Tabriz, Iran; 3 Anesthesiology and Critical Care Department, Iran University of Medical Sciences, Tehran, Iran; 4 Anesthesiology and Critical Care Department, Tabriz University of Medical Sciences, Tabriz, Iran; 5 Students Research Committee, Tabriz University of Medical Sciences, Tabriz, Iran; 6 Neurosciences Research Center, Tabriz University of Medical Sciences, Tabriz, Iran

**Keywords:** Intubation, Emergency Department, Hemodynamic Indices, Alfentanil, Fentanyl, Sufentanil

## Abstract

**Background:**

Laryngoscopy and tracheal intubation lead to the alteration of hemodynamic parameters, including blood pressure and heart rate, in traumatic patients who sustain rapid sequence intubation (RSI). Various drugs such as fentanyl, alfentanil and sufentanil have been used to modify these hemodynamic responses.

**Objectives:**

The aim of the present study is to compare the effects of fentanyl, sufentanil and alfentanil in trauma patients who require RSI in the emergency department (ED).

**Patients and Methods:**

This was a randomized double-blinded study conducted on 90 patients (18-65 years old, ASA I, II), who needed intubation following trauma. The patients were randomly divided into three groups, Group I, Group II and Group III, who have received alfentanil, fentanyl and sufentanil, respectively. Heart rate, blood pressure, saturation of peripheral oxygen and end-tidal carbon dioxide were measured 5 minutes before and 3, 5 and 10 minutes after intubation, respectively. The changes of the hemodynamic parameters were compared in between groups. Data were analyzed by One-way ANOVA, General Linear Model Repeated Measure and Mauchly’s Sphericity Test. A P < 0.05 was considered statistically significant.

**Results:**

There was no significant statistical difference among groups with respect to hemodynamic parameters.

**Conclusions:**

Alfentanil, fentanyl and sufentanil can be used safely as premedication drugs for trauma patients who need intubation.

## 1. Background

In the trauma patient, the priority is airway management (1). The most common method used for tracheal tube insertion in the ED is RSI or direct laryngoscopy. The RSI has been accepted as the most advisable approach for patients who need intubation (2). Also, RSI is the principle of modern airway management in the ED for achieving tracheal tube insertion. However, it is necessary to prescribe simultaneously a potent hypnotic, analgesic and muscle relaxant (2-4). Intubation and direct laryngoscopy can increase heart rate (HR) and blood pressure (BP), a phenomenon described as pressor response (5-7). Tachycardia and hypertension lead to an imbalance between the supply and demand of oxygen to the myocardial muscle, which can lead to ischemia, myocardial infarction and cardiac failure (8). Thus, undesirable hemodynamic responses to intubation should be attenuated via different intubation techniques or pharmacological agents. The laryngoscopic stimulation of the oropharyngolaryngeal structures and the distention of the supraglottic tissues may play an important role in hemodynamic stress response (9). This pressor response is transient, occurring 30 seconds after intubation and lasting for less than 10 minutes. Opioids have been used to block the pressor response (10).

Synthetic opioids, such as alfentanil, sufentanil and fentanyl, are frequently used for adult intubation (6). Comparisons between the numerous effects of these drugs in children have been conducted in various studies, yet for adult patients the data are only limited.

## 2. Objectives

The aim of the present study was to compare the effects of these drugs on the variation of hemodynamic parameters in intubated traumatic patients.

## 3. Patients and Methods

This study was a randomized double-blinded design and it was been carried out during 9 months (from October 2012 to June 2013) in the ED of Imam Reza Research and Training Hospital, Tabriz, East Azerbaijan Province, Iran, with an annual admission rate of 110000 patients (11). Due to the lack of sufficient studies in this field or due to the lack of similar studies in an emergency department, our study was firstly done in the form of a pilot study on 90 patients (three groups of 30 patients). The collection of samples was done from 8 AM. to 6 PM, 7 days a week.

In conformity with the inclusion criteria of the study, the sample included only the trauma patients referred to Imam Reza Hospital, aged 18-65 years old, who had normal BP, needed emergency intubation and were classified as either ASA I or II patients. 

The study excluded patients who needed crush intubation (unresponsive patient or near death), were allergic to lidocaine, were suspect of a difficult intubation based on the physician's clinical judgment (facial anomaly, large mustache, micrognathia, ear and hand anomaly, large incisor teeth) (11), history of malignant hyperthermia or pseudocholinesterase deficiency, pregnancy, multiple failed intubation attempts and intubation maneuver lasting for more than 20 seconds.

This study has received the approval of the Ethics Committee of Tabriz University of Medical Sciences, with the number 2705 and it has also been registered by the Iranian Registry of Clinical Trials (IRCT) under the number of IRCT2012101011067N1. The patients were randomly divided into three groups, as follows:

The existing opioids (alfentanil, fentanyl and sufentanil) were labeled I, II, III in separate syringes prepared in advance (so that in each milliliter of solution in these syringes there existed similar effective drugs). The intubator doctor, who was unaware of the name of the drug, took a ball from a bag with 90 balls (30 balls with No.1, 30 balls with No.2 and 30 balls with No.3) and chose the related syringe based on the obtained balls to use while intubating. In this study, the doctor and the patient were unaware of the opioids’ names, and all the drugs had been put in the intubation trolley in advance by the project responsible. For Group I, alfentanil 500 µg/cc (Janssen Pharmaceutical Company, Beers, Belgium) with a dosage of 20 µg/kg of body weight. In Group II, fentanyl 50 µg/cc (Mylan Pharmaceutical Company, Saint Priest, France) with a dose of 2 µg/kg. In Group III, sufentanil 5 µg/cc (Mylan Pharmaceutical Company, Saint Priest, France) with a dose of 0.2 µg/kg.

All the patients were hydrated during pre-intubation with Ringer serum 10 mL/kg. After preoxygenation and premedication with lidocaine 1.5 mg/kg and alfentanil (20 µg/kg), fentanyl (2 µg/kg), and sufentanil (0.2 µg/kg) based on the group, respectively, anesthesia was induced with etomidate (0.3 mg/kg) and atracurium 0.2 mg/kg (defasciculating dose). Muscle relaxation was achieved by using succinylcholine (1 mg/kg), administered via a peripheral cannula. After 1 minute from the administration of succinylcholine, the trachea was intubated with an appropriate size orotracheal tube.

**Figure 1.**
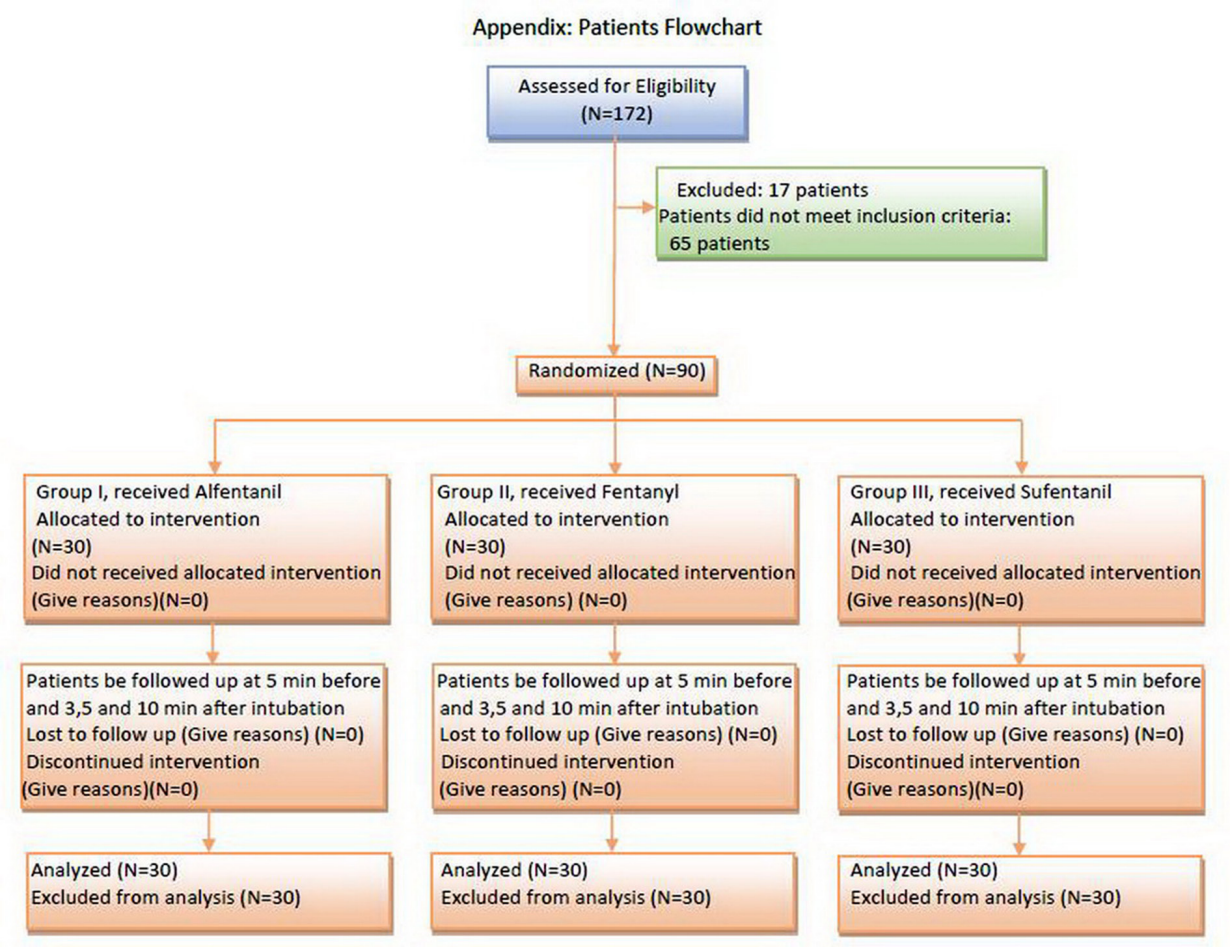
Flow Chart of the Study

Cardiac monitoring was connected for all patients by a monitoring device before intubation (Saadat Novin S1800, Pooyandegan Rah Saadat Corporation, Tehran, Iran). HR, BP and the SpO_2_ were registered by the mentioned device and also, the rate of ETCO_2_ (12) was measured by another monitoring device (NICO 7300, Novametrix Corporation, Wallingford, USA). Five minutes after registering these values, intubation was performed, and in minutes 3, 5 and 10 after intubation, the mentioned indices were registered again. Figure 1 reveals a flow chart of the measurements of these parameters. For data analysis, the SPSS version 17.01 (SPSS Inc., Chicago, Illinois) was used. The normal distribution of the data was surveyed using the Kolmogorov-Smirnoff test.

For the statistical comparison of the hemodynamic parameters among the three groups, One-way ANOVA Test was used. The changes of the hemodynamic parameters in each group were studied using the General Linear Model Repeated Measure and Mauchly’s Test of Sphericity. In all cases, a P < 0.05 was considered statistically significant. 

## 4. Results

A total of 90 patients (71 men and 19 women) participated in this study. The average age of the study group was 41.73 ± 15.3. From the statistical view point, there was no significant difference between the three groups considering age (P = 0.116) and sex (P = 0.282). Table 1 shows sex, ASA class and mean age distribution in all groups. There was no significant statistical difference among groups concerning HR (P = 0.319), Systolic BP (SBP) (P = 0.76), SpO_2_ (P = 0.336), ETCO_2_ (P = 0.111) and Diastolic BP (DBP) (P = 0.24) in the 5 minutes before and the 3, 5 and 10 minutes after intubation (Table 2). 

**Figure 2.**
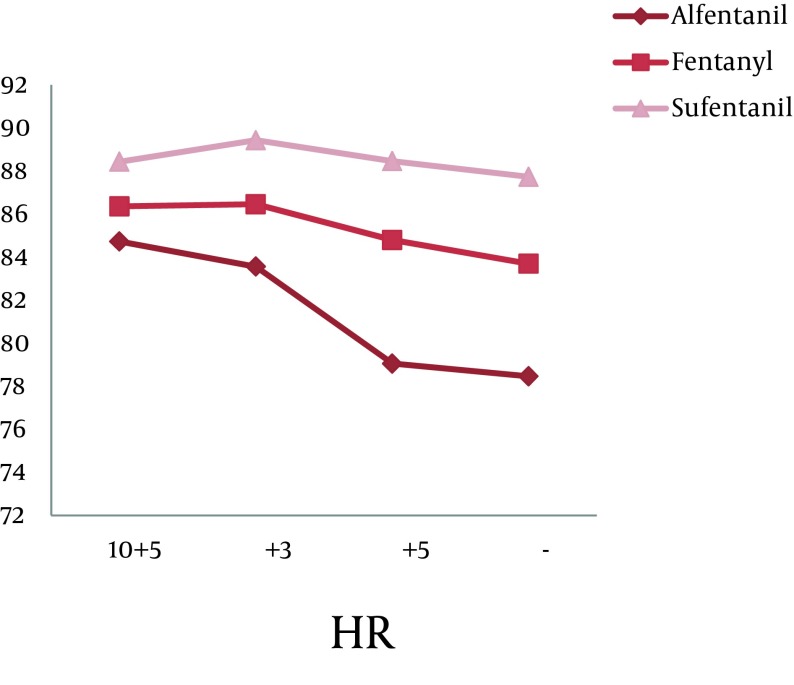
Changes in HR during Repeated Measuring (P < 0.001)

**Figure 3.**
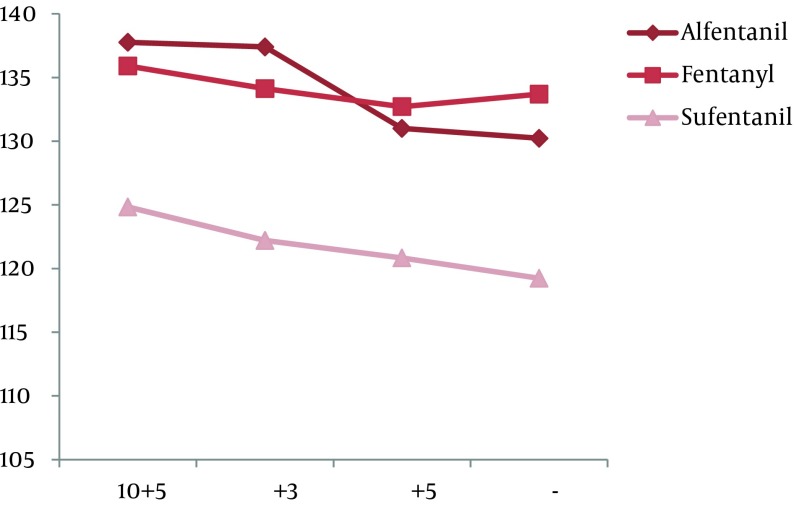
Changes in SBP during Repeated Measuring (P < 0.001)

There was a statistically significant difference within each group during the intubation phase concerning BP (both systolic and diastolic), but with a variation no greater than ± 20%. The repetitive analysis of the variables concerning this study, in relation to the administration of opioids is shown in Figures 2 and 3 (P < 0.001).

**Table 1.** Patients’ Characteristics

Variables	Group I (Alfentanil) (N = 30)	Group II (Fentanyl) (N = 30)	Group III (Sufentanil) (N = 30)
**Age, Mean ± SD, y**	37.06 ± 14.49	44.43 ± 16.08	43.96 ± 14.22
**Sex, Female/Male**	4/26	4/26	9/21
**ASA Class I**	25	22	23
**ASA Class II**	5	8	7

**Table 2.** Pre-Intubation and Post-Intubation Records of SBP and DBP, HR, SpO_2_ and ETCO_2_

Variables	Group I (Alfentanil) (N = 30)	Group II (Fentanyl) (N = 30)	Group III (Sufentanil) (N = 30)
**5 Minutes Before Intubation, mean ± SD**			
HR ^a^	84.73 ± 21.33	86.36 ± 19.54	88.43 ± 18.03
SBP ^a^	137.76 ± 28.50	135.90 ± 25.49	124.83 ± 21.84
DBP ^a^	82.06 ± 17.56	86.46 ± 14.16	80.66 ± 17.86
SpO_2_^a^	83.36 ± 14.81	89.20 ± 7.65	88.90 ± 9.16
ETCO_2_^a^	37.66 ± 6.88	36.06 ± 7.39	34.56 ± 5.81
**3 Minutes After Intubation, mean ± SD**			
HR	83.56 ± 20.67	86.46 ± 19.12	89.43 ± 17.98
SBP	137.40 ± 29.93	134.13 ± 25.62	122.20 ± 21.43
DBP	78.76 ± 16.02	84.76 ± 13.80	78.56 ± 15.35
SpO_2_	95.86 ± 4.65	96.93 ± 2.93	95.70 ± 4.94
ETCO_2_	35.50 ± 4.85	34.63 ± 5.36	32.63 ± 4.76
**5 Minutes After Intubation, mean ± SD**			
HR	79.06 ± 18.91	84.80 ± 15.75	88.46 ± 18.03
SBP	131.00 ± 28.75	132.70 ± 26.39	120.83 ± 20.75
DBP	77.10 ± 15.60	82.13 ± 14.66	78.63 ± 16.85
SpO_2_	97.40 ± 3.90	97.13 ± 3.14	97.26 ± 3.45
ETCO_2_	34.00 ± 4.27	33.46 ± 4.23	32.03 ± 4.20
**10 Minutes After Intubation, mean ± SD**			
HR	78.46 ± 19.05	83.70 ± 16.09	87.73 ± 17.99
SBP	130.23 ± 25.51	133.70 ± 25.25	119.23 ± 21.76
DBP	77.96 ± 12.62	83.03 ± 13.80	76.86 ± 14.24
SpO_2_	97.86 ± 3.54	97.63 ± 3.06	98.20 ± 2.67
ETCO_2_	33.36 ± 3.36	33.50 ± 4.40	32.03 ± 3.93

^a^Abbreviations: DBP, diastolic blood pressure; ETCO_2_, end-tidal carbon dioxide; HR, heart rate; SBP, systolic lood pressure; SpO_2_, saturation of peripheral oxygen.

## 5. Discussion

The use of various drugs (such as hypnotics, opiates and muscle relaxants) during tracheal intubation may lead to the alteration of hemodynamic parameters in patents. In most situations, RSI is the most common approach to control the emergency airway which is used by Emergency Medicine (13-15). Based on the previous evidences, the hemodynamic parameters, before and after intubation by RSI, should not record a variation greater than ± 20%. The drugs used in our study (alfentanil, fentanyl, sufentanil) did not modify the hemodynamic parameters more than 20% (16-24).

Various studies have been conducted to investigate the hemodynamic response following intubation and the hemodynamic parameters changes due to numerous inducing agents. However, all these reports have been conducted mainly on infant-age patients, or in the operating room, in elective conditions. The study of Mireskandari et al. on 80 children aged 1 to 6 years old concluded that fentanyl, in comparison with sufentanil, alfentanil and remifentanil, ensures a superior hemodynamic stability (6). In this study, at three different stages i.e. base line (prior to opioid usage), before laryngoscopy and a minute after intubation, the hemodynamic responses were evaluated. However, in the repeated measuring following intubation, not all parameters were measured. Iftikhar et al., in their study on 60 patients, have concluded that oral gabapentin before intubation leads to the reduction of the stressor response in elective surgeries (8). Safavi et al. in their study on 60 patients, have concluded that, in case of sufficient time for injecting and onset of the effect of opioids before intubation, there is no difference between intravenous pethidine and intravenous sufentanil, in controlling the stressor response following intubation (25). Xue et al. investigated 93 children aged 3 to 9 years old, and have come to conclusion that the injection of sufentanil during intubation leads to a better control of the stressor response following intubation. In this study, various doses of the same medication were administered. In contrast, different agents were used in our study (26). Ko et al. in their study on 90 patients with age greater than 65 years old, have concluded that pretreatment with remifentanil, rather than fentanyl, is more effective in suppressing cardiovascular responses due to endotracheal intubation. However, in our study, remifentanil was not utilized and its comparison with other opioids is suggested (27).

Our results are different from the one reported in the studies of Mireskandari et al. (6), Ko et al. (27) and Xue et al. (26) studies. Our study has been focused on the hemodynamic changes following the intubation of traumatic patients with ASA classification I and II who have normal blood pressure. Since no statistically significant difference was observed in the hemodynamic parameters (SBP, DBP, HR, SpO_2_, and ETCO_2_) of patients, we can use these opioid agents safely in the RSI. However, in the reference books on emergency medicine, fentanyl has been introduced as an opioid in RSI.

According to the results of our study, these three opioids (fentanyl, alfentanil and sufentanil) can be used for intubating trauma patients in the emergency department without significant differences.

### 5.1. Limitations

As time-to-peak effect of these opioids is different, an aspect that has not been taken into consideration in the present study, future investigations should be designed to also assess this different effect. Due to the sample collection, which was done at specific moments of the day (8:00 AM - 6:00 PM), we were limited to include only patients who needed intubation during this time interval. Other limitations of this study were the sample size, which was too small, and also the fact that data were collected in a single center.
